# Supplementary material for: Technical Optimization of SyntheticMR for the Head and Neck on a 3T MR-Simulator and 1.5T MR-Linac: A Prospective R-IDEAL Stage 2a Technology Innovation Report
Source: medRxiv. 2025 Apr 10:2025.04.08.25325491. Preprint. [Version 1] doi: 10.1101/2025.04.08.25325491 (PMC12036405; doi:10.1101/2025.04.08.25325491)
Supplement: Supplement 1 [file media-1.pdf]

Supplementary Materials

MR-Simulator:

Table S1. Echo time and acceleration factor optimization acquired with 50 slices using slice thickness = 3 mm, slice gap = 0.3 mm, TR = 7310 ms, and TI = 173 / 1050 / 3389 / 7190 ms.

GRAPPA Factor	TE_1 / TE_2 (ms)	Acq. Time (mm:ss)	T1 Mean Absolute Bias (%)	T2 Mean Absolute Bias (%)	PD Mean Absolute Bias (%)
1	9.4 / 94	13:28	3.37	43.21	9.12
2	9.4 / 94	7:21	3.21	43.85	9.03
3	9.4 / 94	4:57	3.83	42.91	9.15
4	9.4 / 94	3:59	3.35	41.12	9.41
5	9.4 / 94	3:31	3.70	43.69	7.84
1	19 / 94	13:28	3.60	7.06	14.75
2	19 / 94	7:21	3.74	4.07	16.10
3	19 / 94	4:57	4.30	2.46	17.59
4	19 / 94	3:59	3.26	4.38	15.90
5	19 / 94	3:31	4.65	2.66	16.52
1	28 / 94	13:28	3.13	3.88	18.30
2	28 / 94	7:21	3.10	4.53	18.57
3	28 / 94	4:57	3.17	3.28	18.58
4	28 / 94	3:59	2.34	5.36	18.29
5	28 / 94	3:31	3.52	4.95	19.50
1	9.4 / 113	13:28	3.39	38.15	9.41
2	9.4 / 113	7:21	3.11	38.91	9.21
3	9.4 / 113	4:57	3.52	38.63	9.28
4	9.4 / 113	3:59	2.74	37.58	9.14
5	9.4 / 113	3:31	3.19	43.71	8.38
1	19 / 113	13:28	3.65	7.25	14.85
2	19 / 113	7:21	3.66	7.29	15.40
3	19 / 113	4:57	4.08	5.91	16.89
4	19 / 113	3:59	3.24	7.29	15.23
5	19 / 113	3:31	4.29	7.53	15.72
1	28 / 113	13:28	3.08	2.57	17.62
2	28 / 113	7:21	3.15	2.64	17.80
3	28 / 113	4:57	3.39	1.65	18.39
4	28 / 113	3:59	2.61	3.97	18.02
5	28 / 113	3:31	3.43	3.80	18.48

Table S2. Echo time and acceleration factor optimization acquired with 50 slices using slice thickness = 4 mm, slice gap = 0.4 mm, TR = 7210 ms, and TI = 169 / 1034 / 3341 / 7090 ms.

GRAPPA Factor	TE_1 / TE_2 (ms)	Acq. Time (mm:ss)	T1 Mean Absolute Bias (%)	T2 Mean Absolute Bias (%)	PD Mean Absolute Bias (%)
1	9.4 / 94	13:28	3.48	45.93	17.96
2	9.4 / 94	7:21	3.71	46.18	18.33
3	9.4 / 94	4:57	3.82	44.13	17.95
4	9.4 / 94	3:59	3.39	45.32	18.25
5	9.4 / 94	3:31	4.20	44.95	17.10
1	19 / 94	13:28	3.63	7.95	24.24
2	19 / 94	7:21	3.76	6.59	26.19
3	19 / 94	4:57	3.90	5.50	25.08
4	19 / 94	3:59	3.37	6.44	24.72
5	19 / 94	3:31	4.11	4.51	25.30
1	28 / 94	13:28	3.39	2.92	29.48
2	28 / 94	7:21	3.28	3.65	30.08
3	28 / 94	4:57	3.44	4.91	29.34
4	28 / 94	3:59	2.92	3.33	29.99
5	28 / 94	3:31	3.56	3.55	28.76
1	9.4 / 113	13:28	3.10	40.27	17.69
2	9.4 / 113	7:21	3.27	41.25	18.03
3	9.4 / 113	4:57	3.37	42.04	17.45
4	9.4 / 113	3:59	3.35	41.41	18.13
5	9.4 / 113	3:31	3.38	44.61	16.73
1	19 / 113	13:28	3.66	8.81	24.58
2	19 / 113	7:21	3.71	9.69	24.83
3	19 / 113	4:57	3.64	9.73	24.37
4	19 / 113	3:59	3.21	9.87	24.25
5	19 / 113	3:31	4.11	11.68	24.18
1	28 / 113	13:28	3.23	2.76	28.76
2	28 / 113	7:21	3.34	2.92	29.36
3	28 / 113	4:57	3.44	4.36	28.73
4	28 / 113	3:59	2.78	2.95	28.91
5	28 / 113	3:31	3.31	3.20	27.73

Table S3. Number of slices and TR optimization using slice thickness = 3 mm, slice gap = 0.3 mm, GRAPPA factor = 3, and TE = 19 / 94 ms.

TR (ms)	Slices	TI_1 / TI_2 / TI_3 / TI_4 (ms)	Acq. Time (mm:ss)	T1 Mean Absolute Bias (%)	T2 Mean Absolute Bias (%)	PD Mean Absolute Bias (%)
4390	30	173 / 612 / 2075 / 4270	3:19	3.65	4.24	11.27
4680	32	173 / 758 / 2220 / 4560	3:32	3.62	3.99	11.82
4970	34	173 / 757 / 2219 / 4850	3:45	3.65	3.72	12.04
5270	36	173 / 758 / 2369 / 5150	3:59	3.47	4.10	12.21
5560	38	173 / 758 / 2514 / 5440	4:12	3.52	4.48	12.59
5850	40	173 / 758 / 2659 / 5730	4:25	3.45	3.96	11.89
6140	42	173 / 904 / 2804 / 6020	4:38	3.59	3.68	12.64
6430	44	173 / 903 / 2949 / 6310	4:51	3.29	4.37	12.65
6730	46	173 / 904 / 3099 / 6610	5:04	3.37	3.89	12.87
7020	48	173 / 904 / 3244 / 6900	5:18	3.51	4.18	12.96
7310	50	173 / 1050 / 3389 / 7190	5:31	3.60	4.00	13.67
7600	52	173 / 1050 / 3388 / 7480	5:44	3.23	4.36	14.22
7900	54	173 / 1051 / 3538 / 7780	5:58	3.34	4.15	13.91
8190	56	173 / 1050 / 3863 / 8070	6:11	3.24	5.25	14.17
8480	58	173 / 1050 / 3828 / 8360	6:24	3.34	4.40	14.34
8770	60	173 / 1196 / 3973 / 8650	6:37	3.30	4.63	14.42

Table S4. Turbo factor optimization using 50 slices with slice thickness = 3 mm, slice gap = 0.3 mm, GRAPPA factor = 3, TE = 19 / 94 ms, TR = 7310 ms, and TI = 173 / 1050 / 3389 / 7190 ms.

Turbo Factor	Acq. Time (mm:ss)	T1 LCCC	T2 LCCC	PD LCCC	T1 Mean Absolute Bias (%)	T2 Mean Absolute Bias (%)	PD Mean Absolute Bias (%)
5	6:29	0.9989	0.9965	0.9805	2.52	3.36	11.65
6	5:31	0.9974	0.9951	0.9733	3.47	4.37	13.65

Table S5. Repeatability analysis using 50 slices with slice thickness = 3 mm, slice gap = 0.3 mm, GRAPPA factor = 3, TE = 19 / 94 ms, TR = 7310 ms, TI = 173 / 1050 / 3389 / 7190 ms, turbo factor = 6, and acquisition time = 5:31.

Repeat #	T1 LCCC	T2 LCCC	PD LCCC	T1 Mean Absolute Bias (%)	T2 Mean Absolute Bias (%)	PD Mean Absolute Bias (%)
**1**	0.9976	0.9976	0.9732	3.43	4.17	13.91
**2**	0.9977	0.9965	0.9723	3.25	4.30	14.03
**3**	0.9977	0.9952	0.9732	3.24	4.75	13.93
**4**	0.9974	0.9957	0.9719	3.32	4.88	14.11
**5**	0.9974	0.9951	0.9733	3.47	4.37	13.65
**CoV (%)**	0.01	0.09	0.06	2.77	6.10	1.11

MR-Linac:

Table S6. Image quality optimization. Each acquisition had 60 slices with a slice thickness = 3 mm with a TE = 12 / 120 ms. Note that the slice gap of 0.3 mm with the desired superior-inferior coverage was not feasible at a CS-SENSE acceleration factor of 2. The field-of-view was 284 x 284 mm^2 with an acquired voxel size of 1.00 x 1.00 mm^2 reconstructed to 1.00 x 1.00 mm^2.

Slice Gap (mm)	CS-SENSE Factor	Denoising Factor	Refocusing Flip Angle (°)	SofTone Enabled	TR (ms)	TI_1 / TI_2 / TI_3 / TI_4 (ms)	Acq. Time (mm:ss)
1	2	Weak	120	No	8976	149 / 1196 / 4039 / 8826	6:09
1	2	Weak	120	Yes	8988	149 / 1198 / 4044 / 8838	
1	2	Weak	160	No	9001	150 / 1200 / 4050 / 8850	
1	2	Weak	160	Yes	9014	150 / 1201 / 4056 / 8863	
1	2	Strong	120	No	8976	149 / 1196 / 4039 / 8826	
1	2	Strong	120	Yes	8988	149 / 1198 / 4044 / 8838	
1	2	Strong	160	No	9001	150 / 1200 / 4050 / 8850	
1	2	Strong	160	Yes	9014	150 / 1201 / 4056 / 8863	
0.3	3	Weak	120	No	10772	179 / 1436 / 4847 / 10592	4:30
0.3	3	Weak	120	Yes	10788	179 / 1438 / 4854 / 10608	
0.3	3	Weak	160	No	10803	180 / 1440 / 4861 / 10622	
0.3	3	Weak	160	Yes	10819	180 / 1442 / 4868 / 10638	
0.3	3	Strong	120	No	10772	179 / 1436 / 4847 / 10592	
0.3	3	Strong	120	Yes	10788	179 / 1438 / 4854 / 10608	
0.3	3	Strong	160	No	10803	180 / 1440 / 4861 / 10622	
0.3	3	Strong	160	Yes	10819	180 / 1442 / 4868 / 10638	
1	3	Weak	120	No	8976	149 / 1196 / 4039 / 8826	3:45
1	3	Weak	120	Yes	8988	149 / 1198 / 4044 / 8838	
1	3	Weak	160	No	9001	150 / 1200 / 4050 / 8850	

1	3	Weak	160	Yes	9014	150 / 1201 / 4056 / 8863	
1	3	Strong	120	No	8976	149 / 1196 / 4039 / 8826	
1	3	Strong	120	Yes	8988	149 / 1198 / 4044 / 8838	
1	3	Strong	160	No	9001	150 / 1200 / 4050 / 8850	
1	3	Strong	160	Yes	9014	150 / 1201 / 4056 / 8863	

Table S7. Phantom image quality optimization. Each acquisition had 50 slices with a slice thickness = 3 mm with a TE = 12 / 120 ms. The field-of-view was 284 x 284 mm^2 with an acquired voxel size of 1.00 x 1.00 mm^2 reconstructed to 1.00 x 1.00 mm^2.

Slice Gap (mm)	CS-SENSE Factor	Denoising Factor	Refocusing Flip Angle (°)	SofTone Enabled	T1 Mean Absolute Bias (%)	T2 Mean Absolute Bias (%)	PD Mean Absolute Bias (%)
1	2	Weak	120	No	1.63	9.35	7.51
1	2	Weak	120	Yes	1.94	9.43	7.34
1	2	Weak	160	No	2.68	4.72	3.20
1	2	Weak	160	Yes	2.47	4.84	3.50
1	2	Strong	120	No	2.16	9.80	7.03
1	2	Strong	120	Yes	2.33	9.99	8.06
1	2	Strong	160	No	2.68	5.84	3.18
1	2	Strong	160	Yes	2.89	6.23	3.20
0.3	3	Weak	120	No	3.04	11.15	8.47
0.3	3	Weak	120	Yes	3.18	12.08	8.59
0.3	3	Weak	160	No	5.33	7.81	4.65
0.3	3	Weak	160	Yes	5.99	7.46	4.31
0.3	3	Strong	120	No	3.46	12.77	8.60
0.3	3	Strong	120	Yes	3.23	12.96	8.66
0.3	3	Strong	160	No	6.34	8.91	4.43
0.3	3	Strong	160	Yes	8.12	7.88	4.49
1	3	Weak	120	No	2.08	13.12	8.48
1	3	Weak	120	Yes	2.66	13.78	8.23
1	3	Weak	160	No	2.96	8.26	3.55
1	3	Weak	160	Yes	3.66	8.88	3.68
1	3	Strong	120	No	2.53	14.16	8.01
1	3	Strong	120	Yes	2.49	12.92	7.76
1	3	Strong	160	No	3.37	8.80	3.50
1	3	Strong	160	Yes	3.49	9.18	3.60

Table S8. Echo time optimization. The TR was set to the shortest possible value which is why it varies across acquisitions. Each acquisition had 50 slices with a slice thickness = 3 mm and slice gap = 1 mm, CS-SENSE factor = 2 with strong denoising, refocusing flip angle = 160°, TSE factor = 12, and no SofTone was applied. The field-of-view was 256 x 256 mm^2 with an acquired voxel size of 2.00 x 2.19 mm^2 reconstructed to 1.00 x 1.00 mm^2.

TE_1 / TE_2 (ms)	TR (ms)	Acq. Time (mm:ss)	T1 Mean Absolute Bias (%)	T2 Mean Absolute Bias (%)	PD Mean Absolute Bias (%)
11 / 107	8599	5:53	4.27	9.06	4.31
21 / 108	9199	6:17	4.14	5.67	6.97
12 / 117	8793	6:00	4.75	8.65	3.98
22 / 117	9199	6:17	4.53	4.93	6.46
31 / 118	9798	6:42	4.85	8.23	8.22
13 / 127	9487	6:29	4.90	7.83	3.97
24 / 127	9252	6:19	4.44	4.01	5.86
33 / 127	9798	6:42	4.15	8.58	8.29

Table S9. Repetition time optimization. Each acquisition had a slice thickness = 3 mm and slice gap = 1 mm, CS-SENSE factor = 2 with strong denoising, refocusing flip angle = 160°, TSE factor = 12, and no SofTone was applied. TE = 24 / 127 ms. The field-of-view was 256 x 256 mm^2 with an acquired voxel size of 2.00 x 2.19 mm^2 reconstructed to 1.00 x 1.00 mm^2. Note that the number of slices greater than 50 was not explored due to its excessive scan time compared to our desired 6-minute limitation.

TR (ms)	Slices	TI_1 / TI_2 / TI_3 / TI_4 (ms)	Acq. Time (mm:ss)	T1 Mean Absolute Bias (%)	T2 Mean Absolute Bias (%)	PD Mean Absolute Bias (%)
5551	30	185 / 740 / 2590 / 5365	3:48	4.93	6.54	6.10
5921	32	185 / 925 / 2775 / 5735	4:03	4.66	5.94	5.38
6291	34	185 / 925 / 2775 / 6105	4:18	4.81	6.36	5.56
6662	36	185 / 925 / 2960 / 6476	4:33	4.28	6.56	5.89
7032	38	185 / 925 / 3145 / 6846	4:48	4.77	6.23	5.80
7402	40	185 / 925 / 3330 / 7216	5:04	4.79	5.24	5.65
7772	42	185 / 1110 / 3515 / 7586	5:19	4.39	5.92	5.53
8142	44	185 / 1110 / 3700 / 7956	5:34	4.45	5.41	5.69
8512	46	185 / 1110 / 3885 / 8326	5:49	4.50	5.58	5.89
8882	48	185 / 1110 / 4070 / 8696	6:04	4.77	5.33	5.94
9252	50	185 / 1110 / 4255 / 9066	6:19	4.83	4.45	6.28

Table S10. TSE factor optimization. Each acquisition had a field-of-view was 256 x 256 mm^2 with an acquired voxel size of 2.00 x 2.19 mm reconstructed to 1.00 x 1.00 mm^2 with a slice thickness = 3 mm and slice gap = 1 mm, CS-SENSE factor = 2 with strong denoising, refocusing flip angle = 160°, and TE = 24 / 127 ms with no SofTone applied.

TSE Factor	TR (ms)	Acq. Time (mm:ss)	T1 LCCC	T2 LCCC	PD LCCC	T1 Mean Absolute Bias (%)	T2 Mean Absolute Bias (%)	PD Mean Absolute Bias (%)
12	9252	6:19	0.9864	0.9886	0.9946	4.80	5.32	6.54
14	10398	5:43	0.9819	0.9935	0.9940	5.59	3.60	6.68

Table S11. Each acquisition had a slice thickness = 3 mm and slice gap = 1 mm, CS-SENSE factor = 2 with strong denoising, refocusing flip angle = 160°, TSE factor = 12, and no SofTone was applied. TR = 9252 ms. TE = 24 / 127 ms. The field-of-view was 256 x 256 mm^2 with an acquired voxel size of 2.00 x 2.19 mm^2 reconstructed to 1.00 x 1.00 mm^2 in an acquisition time of 6:19.

Repeat #	T1 LCCC	T2 LCCC	PD LCCC	T1 Mean Absolute Bias (%)	T2 Mean Absolute Bias (%)	PD Mean Absolute Bias (%)
**1**	0.9876	0.9836	0.9947	4.37	4.88	6.32
**2**	0.9884	0.9792	0.9941	4.41	5.68	6.01
**3**	0.9868	0.9848	0.9948	4.86	5.43	6.18
**4**	0.9863	0.9847	0.9951	4.64	5.26	6.04
**5**	0.9889	0.9797	0.9949	4.23	5.56	6.20
**CoV (%)**	0.10	0.25	0.02	4.99	5.18	1.82
